# Biosynthetic Gas Vesicles from *Halobacteria NRC-1*: A Potential Ultrasound Contrast Agent for Tumor Imaging

**DOI:** 10.3390/pharmaceutics14061198

**Published:** 2022-06-03

**Authors:** Mingjie Wei, Manlin Lai, Jiaqi Zhang, Xiaoqing Pei, Fei Yan

**Affiliations:** 1 Department of Ultrasound, Sun Yat-sen University Cancer Center, State Key Laboratory of Oncology in South China, Collaborative Innovation Center for Cancer Medicine, Guangzhou 510060, China; weimj@sysucc.org.cn; 2 Department of Ultrasound, The First Affiliated Hospital of Shenzhen University, Shenzhen 518061, China; 1910244009@email.szu.edu.cn; 3 Department of Ultrasound, The First Affiliated Hospital, Guangzhou University of Chinese Medicine, Guangzhou 510405, China; 20191109183@stu.gzucm.edu.cn; 4 Center for Cell and Gene Circuit Design, CAS Key Laboratory of Quantitative Engineering Biology, Shenzhen Institute of Synthetic Biology, Shenzhen Institute of Advanced Technology, Chinese Academy of Sciences, Shenzhen 518055, China

**Keywords:** ultrasound contrast agents, gas vesicles, tumor imaging, tumor’s ischemic region

## Abstract

Ultrasound contrast agents are valuable for diagnostic imaging and drug delivery. Generally, chemically synthesized microbubbles (MBs) are micro-sized particles. Particle size is a limiting factor for the diagnosis and treatment of many extravascular diseases. Recently, gas vesicles (GVs) from some marine bacteria and archaea have been reported as novel nanoscale contrast agents, showing great potential for biomedical applications. However, most of the GVs reported in the literature show poor contrast imaging capabilities due to their small size, especially for the in vivo condition. In this study, we isolated the rugby-ball-shaped GVs from *Halobacteria NRC-1* and characterized their contrast imaging properties in vitro and in vivo. Our results showed that GVs could produce stable and strong ultrasound contrast signals in murine liver tumors using clinical diagnostic ultrasound equipment at the optimized parameters. Interestingly, we found these GVs, after systemic administration, were able to perfuse the ischemic region of a tumor where conventional lipid MBs failed, producing a 6.84-fold stronger contrast signal intensity than MBs. Immunohistochemistry staining assays revealed that the nanoscale GVs, in contrast to the microscale MBs, could penetrate through blood vessels. Thus, our study proved these biosynthesized GVs from *Halobacterium NRC-1* are useful for future molecular imaging and image-guided drug delivery.

## 1. Introduction

Ultrasound is one of the leading medical imaging modalities for diagnosis and image-guided therapy due to its numerous advantages such as noninvasiveness, nonradiation, low cost, high spatiotemporal resolution, soft tissue penetration, etc. [1]. With the emergence of ultrasound contrast agents (UCAs), ultrasound is playing more and more important roles in clinical application and pre-clinical medical research. To date, several UCAs are approved for clinical use as diagnostic agents, and researchers are exploring their potential for drug delivery by taking advantage of their cavitation effects [2]. Conventionally, UCAs are lipid, protein, or polymer-shelled microbubbles, with gas cores ranging from 1 to 10 μm in size [3], approximately corresponding to the size of a red blood cell. Leaky vessels with holes (ranging from 200 nm to 1.2 μm) could be formed due to a lack of blood supply in tumor tissues, while the size of gaps between endothelial cells of the fenestrated capillary is about 50 nm [4]. Thus, these microbubbles are restricted within the vasculature [5,6], and it is difficult for them to reach the extravascular diseased tissues, making them unsuitable for molecular imaging applications to target receptors in tumor cells or produce direct cavitation on tumor cells for drug delivery [7]. Although several studies have reported chemically synthesized nanoscale contrast agents with good acoustic properties [8,9,10], there are still many difficulties that need to be solved, including the sophisticated preparation procedure, unfriendly biocompatibility, and unstable structure [11].

In recent years, Shapiro et al. introduced novel biosynthetic nanobubbles as UCAs on the basis of genetically encoded gas vesicles (GVs) [12]. GVs are gas-filled and protein-shelled nanobubbles, with Gvp A consisting of the main vesicle structure and Gvp C binding to the exterior surface. These GVs are formed from marine bacteria and archaea, providing these microorganisms with buoyancy and allowing them to migrate to optimal living conditions [13,14,15]. Unlike chemically synthesized UCAs with a spherical shape, GVs are cylindrical or biconical organelles that are 45–250 nm wide and 100–600 nm long [16], depending on different hosts. Moreover, the 2 nm protein shell of GVs permits surrounding gas to move freely in and out of the vesicle [13], which leads GVs to be inherently stable. However, owing to the small particle size, the contrast imaging effects of GVs are limited for in vivo imaging applications. Many previous reports focused on chemically modifying cylindrical GVs from archaea to lengthen their duration in circulation, or developing novel imaging methods for improving their detection sensitivity [17,18]. Additionally, previous imaging experiments were conducted in animal ultrasound imaging systems with a high-frequency transducer (over 18 MHz), and few studies in the literature reported their successful application using clinical ultrasound diagnostic equipment [19,20]. In the present study, we focused on the ultrasound contrast imaging property of rugby-ball-shaped GVs from *Halobacteria NRC-1* and compared the perfusion abilities of GVs and conventional UCAs in tumor tissues.

## 2. Materials and Methods

### 2.1. Isolation of Gas Vesicles

*Halobacteria NRC-1* (*Halo*) was cultured in an ATCC medium at 37 °C on a shaking incubator at 220 rpm/min. Gas vesicles (GVs) were isolated from *Halo* through lysis, with TMC buffer and centrifugation at 350× *g* for 4 h. The isolated GVs were washed with phosphate-buffered saline (PBS) and further purified three times via centrifugal flotation at 250× *g* for 4 h and stored at 4 °C. The concentration of GVs was determined using a microplate reader (Multiscan GO, Thermo Scientific, Waltham, MA, USA) at 500 nm wavelengths.

### 2.2. Preparation of Lipid Microbubbles

Lipid microbubbles (MBs) were fabricated according to the previous study [21]. Briefly, DSPE-PEG_2000_ and DSPC were dissolved in chloroform with a molar ratio of 1:9 in a tube. A uniform film was formed on the tube wall by pumping nitrogen into the tube to remove the chloroform, followed by vacuum treatment for 2–4 h. The film was hydrated with 5 mL Tris buffer (pH 7.4) consisting of 80% 0.1 M Tris, 10% 1,2-propylene glycol, and 10% glycerol at 60 °C in an ultrasonic cleaner at 40 kHz until the film was completely dissolved. The solution was divided into 5 penicillin bottles (1 mL each bottle) and sealed. The ventilation device was filled with C_3_F_8_ gas to displace the gas in the bottle. MBs were obtained via vibration for 30 s. The resulting MBs were rinsed with PBS three times via the centrifugal flotation method at 20× *g* for 4 min.

### 2.3. Characterization of GVs and MBs

GVs or *Halo* solution was diluted and placed on copper mesh, negatively stained with 2% phosphotungstic acid, then dried at room temperature. The morphologies of GVs were observed by TEM (Hitachi H-7500, Hitachi Limited, Tokyo, Japan). The particle size and zeta potential of GVs and MBs were measured using a Zetasizer analyzer (Zetasizer Nano S90, Malvern, Worcestershire, UK). All samples were diluted to appropriate concentrations at room temperature. The particle size and zeta potential of each sample were measured three times.

### 2.4. In Vitro Ultrasound Imaging

The in vitro ultrasound imaging capability of GVs was determined at different concentrations and mechanical indices (MIs). As a blank control, PBS was used in these experiments. GVs at OD_500_ 1.0–3.0 were added into 1% agarose phantom wells (200 μL each sample). Imaging was performed using the L11-3U line array transducer ultrasound diagnostic equipment (Mindray Resona 7, Mindray, Shenzhen, China) in the contrast imaging mode. The mechanical index (MI) was kept at 0.085. As for the imaging capability of GVs at different mechanical indices (MIs), GVs at OD_500_ 1.5 were used, and MI was changed from 0.064 to 0.281.

### 2.5. In Vivo Ultrasound Imaging

Animal experiments were conducted under protocols approved by the Ethics Committee of Shenzhen Institutes of Advanced Technology, Chinese Academy of Sciences. As for liver imaging, the male C57BL/6 mice (four weeks old, 13–15 g) were maintained with isoflurane anesthesia on a heating pad. GVs at OD_500_ 1.0–3.0 were successively intravenously injected into the mice and imaged with a line array transducer of Mindray Resona 7 in contrast mode. The MI was kept at 0.149. A volume of 150 μL GVs for each dose was injected, and images were continuously acquired for 3 min. At least 15 min elapsed before the next GV injection. With the final injection of GVs at OD_500_ 3.0, burst pulses were applied to collapse the GVs 15 min after injection. As for tumor imaging, tumor-bearing in C57BL/6 male mice (four weeks old, 13~15 g) was developed with subcutaneous injection with 2 × 10^5^ MB49 cells in PBS (50 μL). When the tumor size reached about 200 mm^3^, a volume of 150 μL GVs at OD_500_ 3.0 and MBs (1.09 × 10^8^/mL), respectively, was intravenously injected into the mice, and the tumors were imaged using the equipment as described above. Images were acquired continuously for 15 min until the contrast signals disappeared, before the next injection of lipid MBs. The contrast signals were quantitatively analyzed using the software built in the Mindray Resona 7 device after manually defining the regions of interest (ROI).

### 2.6. Histological Examination

In order to confirm that GVs were able to pass through the endothelial gaps in tumors, we used a fluorescent inverted microscope to observe DiI-dyed GVs in the tumor section. A mixture of DiI-labeled GVs and FITC-labeled lipid MBs were intravenously injected into the tumor-bearing mice, and the contrast imaging signals were observed in tumors using an ultrasound imaging system. When the contrast signals reached their peak, the mice were sacrificed and the tumors were immediately removed for sectioning into 10 μm slices. Slices were incubated with rat anti-mouse CD31 antibody (Abcam, Cambridge, UK) overnight at 4 °C, then incubated with Cy5-conjugated goat anti-rabbit secondary antibody (Abcam, Cambridge, UK) to visualize the vessels. The cell nuclei were stained with DAPI (Sigma Aldrich, St. Louis, MO, USA). Images were recorded using a fluorescent Inverted microscope (IX73, Olympus, Tokyo, Japan).

### 2.7. Toxicity Assay

Briefly, 200 μL GVs at OD_500_ 3.0 were intravenously injected into the C57BL/6 mice. PBS was used as the blank control. The major organs (heart, liver, spleen, lung, and kidney) of mice were taken for H&E staining after 24 h.

### 2.8. Statistical Analysis

The data are expressed as the mean ± standard deviation of the mean. Statistical Product and Service Solution (SPSS) 25.0 was used for statistical analysis. The data were compared using Bonferroni’s test. The significance level was set at *p* < 0.05.

## 3. Results

### 3.1. Characterization of Halo and GVs

**Figure 1:**
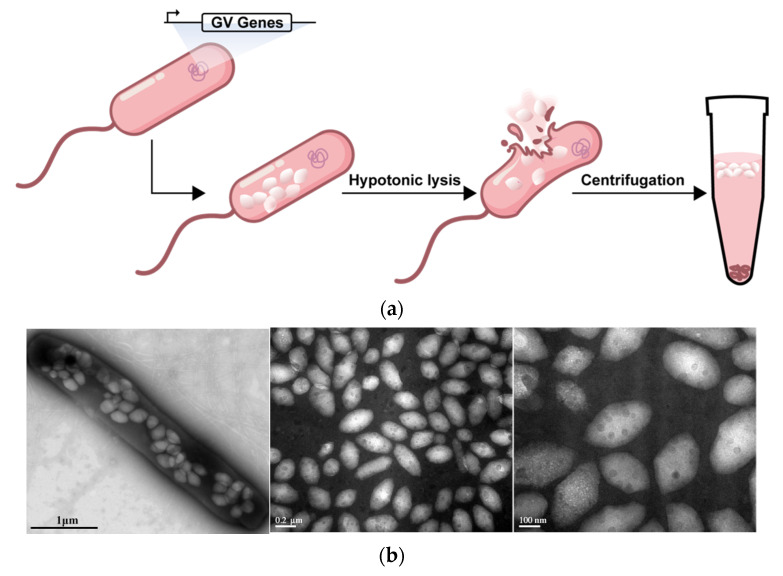

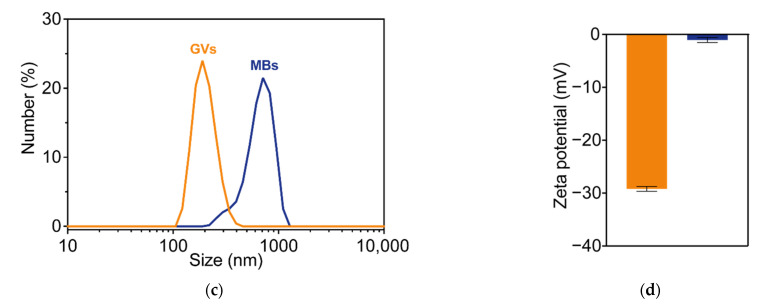
**Preparation and characterization of GVs:** (**a**) diagram of preparation of biosynthetic GVs; (**b**) TEM images of *Halo* (left) and GVs (middle and right); (**c**) particle size distribution of GVs and lipid MBs; (**d**) zeta potential of GVs and lipid MBs. Error bars represent ± SD.

In this study, gene-encoded GVs were biosynthesized in *Halobacterium NRC-1* (*Halo*) and were purified according to the previous protocol (Figure 1a) [22]. Approximately 30-to-40 GVs could be observed in a bacterium, just as shown in the transmission electron microscopy (TEM) image (Figure 1b, left panel). The isolated GVs appeared uniformly rugby-ball-shaped, with 200 nm width and 400 nm length (Figure 1b, middle and right panels). The mean particle size of GVs determined by a Zetasizer analyzer was 224.6 ± 2.3 nm, significantly smaller than lipid microbubbles (MBs, 738.4 ± 51.5 nm) (Figure 1c). Additionally, GVs had −27.8 ±1.6 mV of zeta potential, lower than lipid MBs (Figure 1d). The smaller particle size makes it possible for GVs to pass through the vasculature and reach the extravascular tissues. Meanwhile, the negative zeta potential of GVs is helpful for remaining relatively stable or preventing aggregation.

### 3.2. In Vitro Ultrasound Imaging of GVs

**Figure 2:**
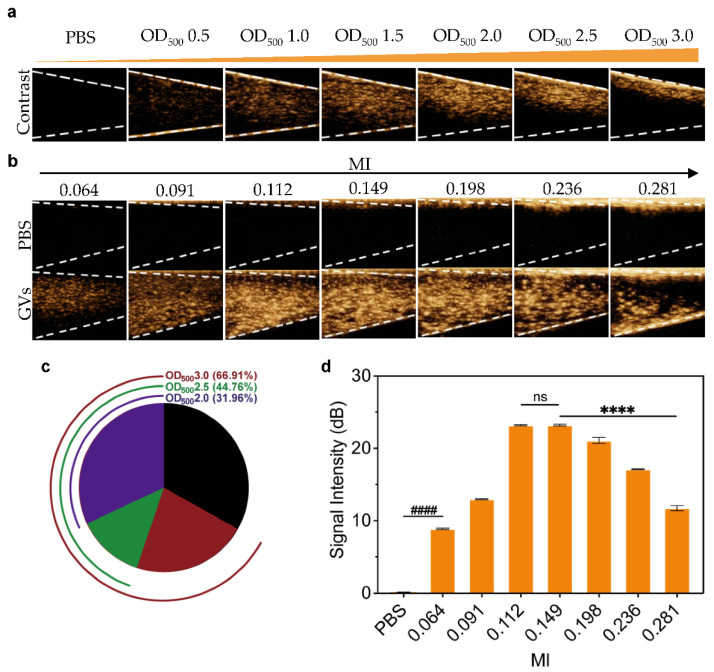
**Ultrasound contrast imaging of GVs in vitro:** (**a**) nonlinear contrast images acquired by a linear array transducer of GVs at different concentrations (OD_500_ 0.5–3.0); (**b**) nonlinear contrast images of 200 μL GVs at OD_500_ 1.5 and 200 μL PBS, when the MI was changed from 0.064 to 0.281; (**c**) the percentage of acoustic attenuation area produced by GVs at OD500 2.0 (purple), OD500 2.5 (green), and OD500 3.0 (red); (**d**) contrast signal intensity produced by GVs at different MI. Error bars represent ± SD. **** *p* < 0.0001. ^####^ *p* < 0.0001. ns, no statistical significance.

Contrast-enhanced ultrasound imaging has been widely used in clinical practice, due to the background-deduced harmonic signals produced by the nonlinear oscillation of UCAs [23,24]. In particular, there could be more nonlinearities, even at lower acoustic pressures, when UCAs are formed with a flexible shell [25]. Shapiro et al. found that substantial second- and third-harmonic signals were observed in GVs from *Halo* in response to 6 MHz transmitted pulses, and GVs from *Halo* produced robust contrast using a scanning single-element ultrasound imaging system operating at 4.8, 8.6, and 17 MHz in vitro [12]. In the present study, we first examined the imaging capability of GVs in gel phantoms using a clinical line array transducer with a frequency between 5.6 and 10.0 MHz. The echo signals of GVs were detectable at GV concentrations from OD_500_ 0.5 to OD_500_ 3.0, and the enhanced contrast signals increased with the increase in GV concentration. However, acoustic attenuation occurred at higher concentrations (OD_500_ > 2.0), showing a reduced signal behind the GVs in the gel phantom (Figure 2a). The acoustic signal attenuation area was about 31.96% at OD_500_ 2.0, 44.76% at OD_500_ 2.5, and 66.91% at OD_500_ 3.0, respectively (Figure 2c). Furthermore, we determined their contrast imaging properties by using ultrasound of different mechanical indices (MIs) while keeping the concentration of GVs at OD_500_ 1.5. In Figure 2b,d, we can see that contrast signals of GVs at all tested concentrations were significantly stronger than the PBS buffer control (*p* < 0.0001). With the increase in MI, the mean signal intensity at the ROI increased when MI was changed from 0.064 to 0.149, reaching 23.21 ± 0.1 dB at MI = 0.149. However, the mean signal intensity decreased when MI continued to increase, with only 11.73 ± 0.3 dB at MI = 0.281 (*p* < 0.0001) (Figure 2d), showing a parabolic trend at MI ranging from 0.064 to 0.281. A possible reason for this may be attributed to over-threshold MI, leading to the collapse of the GVs proximal to the transducer.

### 3.3. In vivo Ultrasound Imaging of GVs

**Figure 3:**
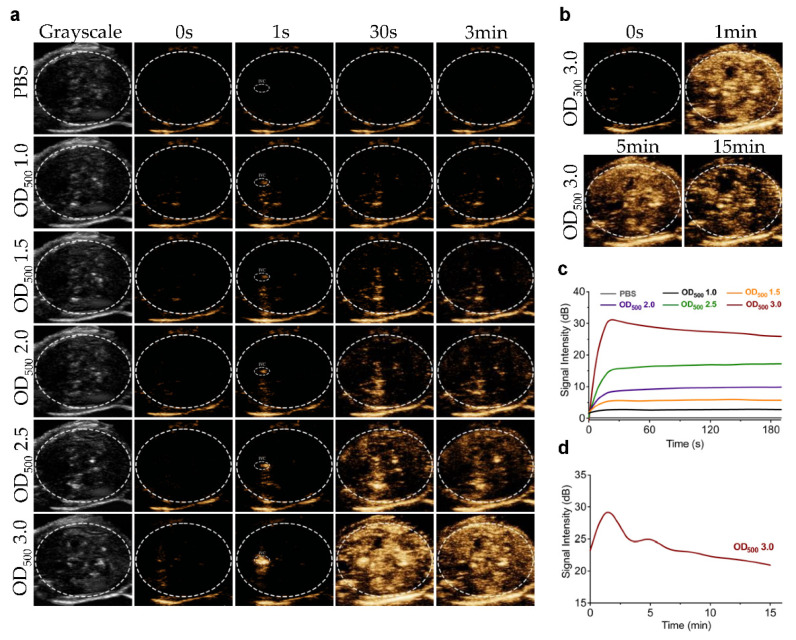
**Ultrasound contrast imaging of GVs in vivo:** (**a**) nonlinear contrast images of GVs (OD_500_ 1.0–3.0) at different time points after tail injection; (**b**) nonlinear contrast images of GVs at OD_500_ 3.0; (**c**) time–intensity curves of GVs at different concentrations in liver within 3 min; (**d**) time–intensity curves of GVs at OD_500_ 3.0 in liver within 15 min.

**Figure 4:**
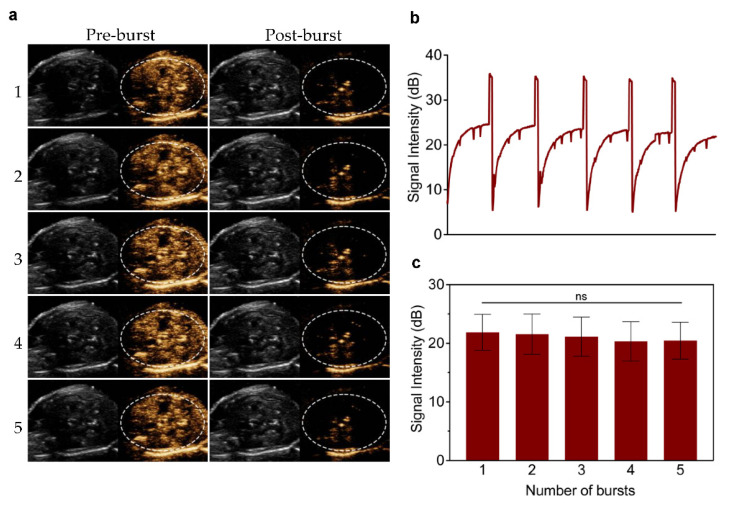
**Re-perfusion of GVs after multiple ultrasonic bursts in vivo:** (**a**) B-mode and nonlinear contrast images of GVs’ re-perfusion process after five ultrasonic bursts, about 30 s between two bursts (**b**) time–intensity curve of contrast signals of GVs during five ultrasonic bursts; (**c**) contrast signal intensities produced by the re-perfusion of GVs after each burst. Error bars represent ± SD. ns, no statistical significance.

To test the contrast imaging capability of GVs in vivo, we injected different concentrations of GVs or PBS into the tail vein of C57BL/6 mice and imaged the liver in B-mode and contrast mode. Compared with the PBS control, we found no apparent contrast signal enhancement in the liver at OD_500_ 1.0. With the increase in the concentrations of GVs, contrast enhancement signals increased and stayed stable for 180 s (Figure 3a,c). The most significantly enhanced signals were produced in the liver at an OD_500_ 3.0 concentration, reaching about 28.16 ± 1.67 dB of contrast signal intensity in the liver and sustaining for more than 15 min (Figure 3b,d). No significant signal enhancement was observed in the liver of mice injected with the PBS control, and no grayscale signal difference was found in all tested doses of GVs in the liver. To further confirm that the contrast signals were produced from GVs, we applied a short, high-power ultrasonic pulse to collapse these GVs. Figure 4a clearly shows these contrast signals disappeared immediately after the high-power burst and gradually reappeared in the liver, reaching a comparable contrast signal intensity before the burst. Interestingly, the disappearance and reappearance of contrast signals could be observed multiple times (at least five times) when the high power bursts were repeated, confirming that the contrast signals really resulted from the GVs. Quantitative analysis of these contrast signals revealed that there were no significant differences before and after burst within five applications, indicating the concentration of GVs in the circulation apparently did not change during this period (Figure 4b,c).

### 3.4. Imaging of Tumor’s Ischemic Zone by GVs

**Figure 5:**
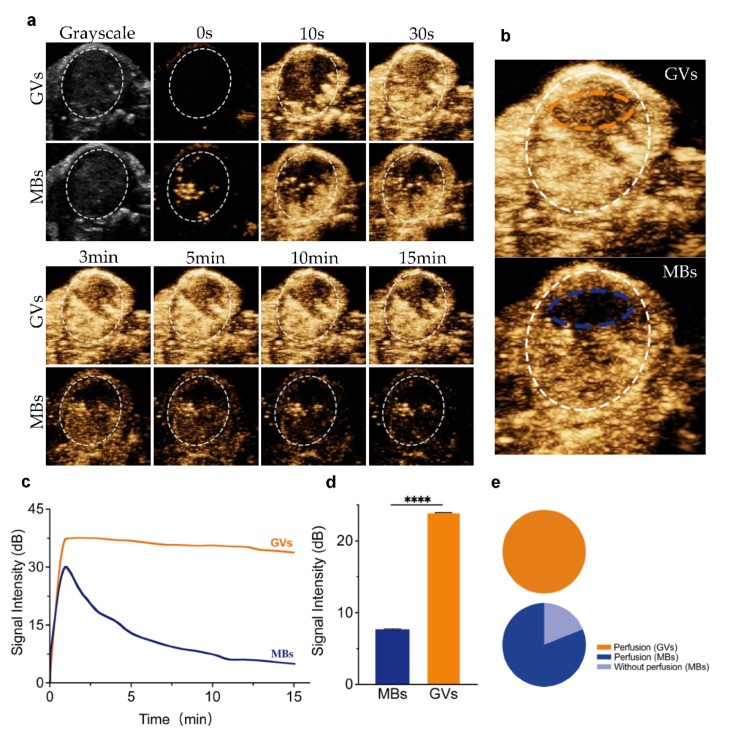
**Ultrasound imaging of GVs and lipid MBs in tumor:** (**a**) B-mode and nonlinear contrast images of GVs and MBs in 15 min after tail injection; (**b**) nonlinear contrast images of the ischemic zone of a tumor after injection of GVs (orange-dotted circle) or MBs (blue-dotted circle); (**c**) time–intensity curve of GVs and MBs perfused into the tumor tissue; (**d**) contrast signal intensities of GVs and MBs in the tumor’s ischemic zone (orange- and blue-dotted area); (**e**) the perfusion area of GVs and MBs in the tumor. Error bars represent ± SD. **** *p* < 0.0001.

Next, considering the nanoscale particle size of GVs, we hypothesized that such nanobubbles were able to perfuse into the tumor’s ischemic zone where conventional microscale UCAs cannot. To demonstrate this, we prepared lipid MBs and removed the smaller nanobubbles in the formulation of MBs by gradient centrifugation [21]. Thus, lipid MBs with a 738.4 ± 51.5 nm mean particle size and −0.5 ± 1.2 mV zeta potential were obtained (Figure 1c,d). Then, MBs and GVs were injected into the tail vein of the same tumor-bearing mice at 30 min intervals, and tumors were subsequently imaged in the B- and contrast modes. Robust contrast signals were observed at the periphery of the tumor after injecting GVs, and GVs gradually spread into the center of the tumor, where the contrast signals decreased more quickly. However, no contrast signals were observed in the center of the tumor after injecting MBs (Supplementary Materials). From Figure 5a, we can see that the injection of GVs could keep the enhanced contrast signals for a longer time after injection (15 min), but with the injection of MBs, the signals only lasted for less than 10 min. The signal intensity of the perfusion tumor area (white-dotted circles) achieved 35.49 ± 1.66 dB for GVs and 31.5 ± 0.72 dB for MBs, respectively (Figure 5c). It is notable that most of the tumor regions were enhanced with contrast signals after injection of MBs or GVs, but only the center of the tumor (ischemic zone) was enhanced after injection of GVs rather than MBs (Figure 5b, orange-dotted circle and blue-dotted circle). These results clearly show that GVs but not MBs can perfuse into the tumor’s ischemic zone. Quantitative analysis revealed that the enhanced contrast signal intensity of the tumor’s ischemic zone (Figure 5b, orange-dotted circle and blue-dotted circle) achieved 20.94 ± 3.48 dB, about 6.84-fold higher than MBs at the same location (Figure 5d). In other words, about 20% of the sectional area could not be perfused by lipid MBs, but GVs could perfuse the whole area (Figure 5e).

### 3.5. Distribution of GVs in Tumor

**Figure 6:**
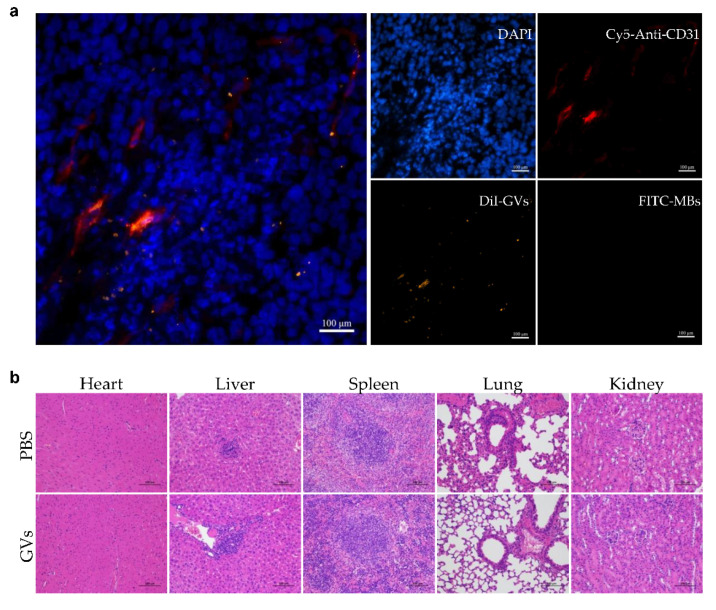
(**a**) Inverted fluorescent microscope images of tumor sections after nuclear and vessel labeling. Red stands for CD31 stained vessels, brown for DiI labeled GVs, and blue for DAPI stained nuclei. The scale bar is 100 μm; (**b**), H&E staining of major organs (heart, liver, spleen, lung, and kidney) of mice treated by GVs at OD_500_ 3.0 or PBS.

To confirm the distribution of GVs and MBs into the tumor’s ischemic zone, the DiI-labeled GVs and FITC-labeled MBs were simultaneously injected into the tail vein of the tumor-bearing mice. The tumors were examined after 30 min, sectioned, and observed with an inverted fluorescent microscope. In Figure 6a, we can see that DiI-labeled GVs appeared not only in the vasculature but also in the extravascular space of the tumor tissues. By contrast, there were no FITC-labeled lipid MBs which could be observed in the tumor section (Figure 6a). The in vivo cytotoxicity also showed that no structural abnormalities were observed in the major organs (heart, lung, liver, spleen, and kidney) in H&E-stained slices after being intravenously injected GVs at OD_500_ 3.0 (Figure 6b). Thus, compared to the lipid MBs, GVs were superior in penetrating tumors, allowing them to achieve contrast imaging capability in a tumor’s ischemic zone.

## 4. Discussion

UCAs are not only key agents for ultrasound contrast-enhanced imaging but also important carriers for drug delivery [2,26]. In recent decades, numerous microscale UCAs such as MBs have been developed and have shown promising prospects in biomedical applications. However, their large particle size (over 1 micron) greatly limits their applications in many extravascular diseased tissues. In this study, we isolated nanoscale GVs from *Halobacterium NRC-1* and confirmed their applicability as UCAs in vitro and in vivo. Unlike chemically synthesized UCAs, GVs were encoded with gas vesicle protein-encoding genes (gvp) and produced in cells, which provided GVs with possible structure modification through gene engineering technology. For example, the acoustic pressure tolerance of GVs can be regulated by combining gvp genes from different microorganism species [27]. Additionally, the cellular origin endows GVs with better biocompatibility for biomedical applications. Studies have reported *Halo* cells do not contain lipopolysaccharide (LPS), making it possible to obtain endotoxin-free GVs [28]. Although our primary results showed that GVs did not cause acute toxicity in mice, the potential adverse events of this contrast agent still need to be investigated. For instance, the immune response should be studied since GVs are made of the protein shell.

The in vitro and in vivo imaging data demonstrated that GVs could produce stable contrast signals within clinical ultrasound diagnostic equipment. Unlike in previous reports, we provided real-time in vivo imaging data of GVs and proved their long duration (over 15 min) in circulation with clinical ultrasound diagnostic equipment, without any modifications to imaging methods. A possible reason for this may be the larger particle size of GVs used in our study than those from *Anabaena flos-aquae* or gene-engineered bacteria. The rugby-balled shape, rather than rod-like shape, of our GVs may also help them to better produce acoustic oscillation responding to ultrasound excitation. The in vivo contrast imaging signals of GVs disappeared after applying a short, high-power burst acoustic pulse, via the flush button built into the ultrasound diagnostic equipment (Supplementary Materials). Additionally, the contrast imaging signals of GVs could be observed again after the reperfusion of GVs, similar to conventional MBs. This might be very beneficial for image-guided drug delivery in vivo when using GVs as the gene or drug carriers [29].

It is worth mentioning that GVs may perfuse into the tumor’s ischemic zone and produce contrast signals, thanks to their nanoscale size, while no signals produced by the microscale MBs were observed in the same area. Additionally, we found that contrast signals of GVs in the tumor’s ischemic zone appeared slower and faded faster than in the surrounding region. It is possible that the leaky tumor vasculature allowed smaller GVs to penetrate the tumor’s ischemic zone but the high interstitial fluid pressure (IFP), which can reach approximately 10~40 mmHg, also helped to wash out these GVs. Although it may partly explain the perfusion contrast imaging of GVs in the tumor’s ischemic zone, further studies using other methods and technologies are still necessary to help confirm this hypothesis. Super-resolution ultrasound imaging [30], for instance, can track the displacement of the UCAs with subwavelength resolution and reconstruct the vascular and velocity maps. Meanwhile, novel ultrasound technologies have been used to identify microvascular morphology features of tumor angiogenesis [31]. At present, researchers must pay attention to the role of tumor microenvironments in tumor therapy [32]. Combining these novel technologies with GVs would greatly broaden the biomedical applications of GVs. In addition, GVs can pass through endothelial gaps, which makes this contrast agent different from the commonly used SonoVue. GVs have potential clinical implications such as diagnosis of some tumors that lack blood supply and delivery of drugs or genes into the ischemic zone.

## 5. Conclusions

In this study, we successfully isolated nanoscale GVs from *Halobacterium NRC-1* and optimized their contrast imaging parameters in vitro and in vivo. GVs could produce robust ultrasound contrast signals when MI ranged from 0.112 to 0.149. In other words, our data proved that GVs from *Halobacteria NRC-1* can be used as a promising novel contrast agent for ultrasonography and can be detected by clinical ultrasound diagnostic equipment. Importantly, our results showed these GVs can perfuse and produce strong contrast signals in a tumor’s ischemic zone, in which conventional UCAs were not observed. In conclusion, our study proved these biosynthesized GVs from *Halobacterium NRC-1* are useful for future molecular imaging and image-guided drug delivery.

## Data Availability

Not applicable.

## Supplementary Materials

The following supporting information can be downloaded at: https://www.mdpi.com/article/10.3390/pharmaceutics14061198/s1, Video S1: Reperfusion of GVs after acoustic pulses; Video S2: Tumor imaging of GVs; Video S3: Tumor imaging of MBs.
