# Supplementary figures and images for: Biosynthetic Gas Vesicles from Halobacteria NRC-1: A Potential Ultrasound Contrast Agent for Tumor Imaging
Source: Pharmaceutics. 2022 Jun 3;14(6):1198. doi: 10.3390/pharmaceutics14061198 (PMC9229964; doi:10.3390/pharmaceutics14061198)

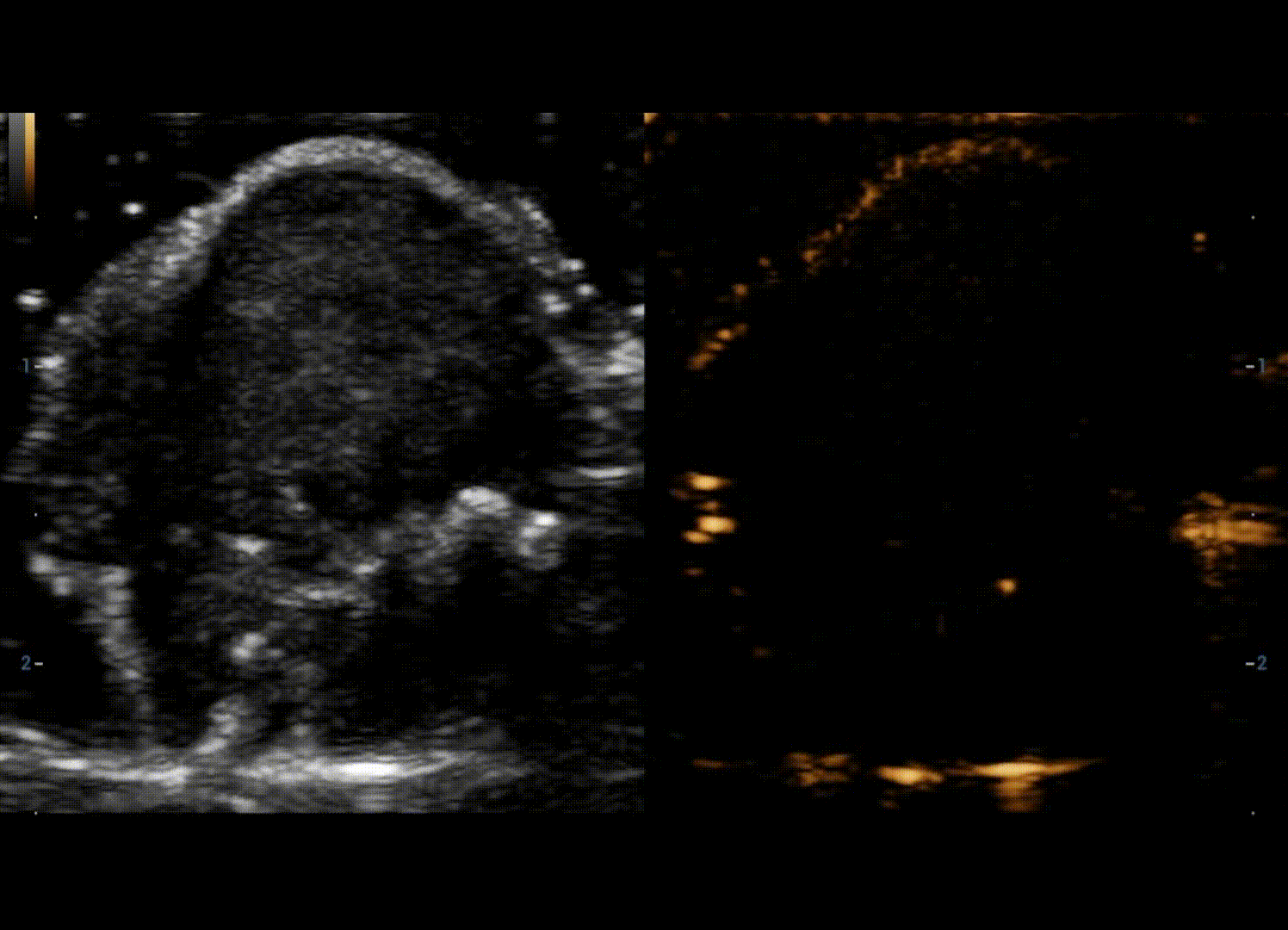

Supplement: Supplementary file 1 [file pharmaceutics-14-01198-s001.zip › Supplementary materials/Video S2. Tumor imaging of GVs.gif]

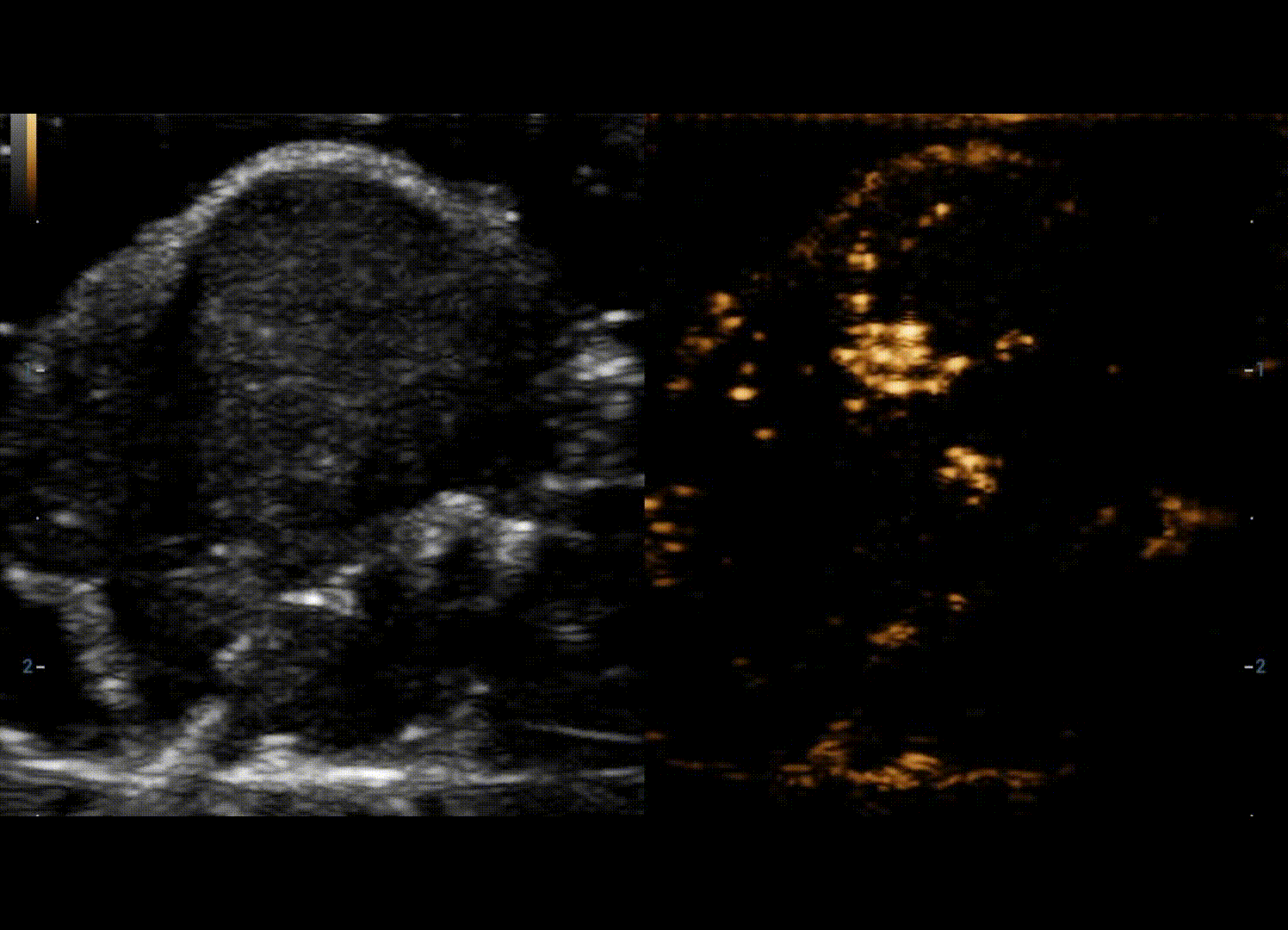

Supplement: Supplementary file 1 [file pharmaceutics-14-01198-s001.zip › Supplementary materials/Video S3. Tumor imaging of MBs.gif]
